# Long-term aspirin use and colorectal cancer risk: a cohort study in Sweden

**DOI:** 10.1038/sj.bjc.6603442

**Published:** 2006-10-24

**Authors:** S C Larsson, E Giovannucci, A Wolk

**Affiliations:** 1 Division of Nutritional Epidemiology, The National Institute of Environmental Medicine, Karolinska Institutet, Stockholm, Sweden; 2 Departments of Nutrition and Epidemiology, Harvard School of Public Health, Boston, MA, USA; 3 Channing Laboratory, Department of Medicine, Brigham and Women's Hospital and Harvard Medical School, Boston, MA, USA

**Keywords:** aspirin, cohort studies, colorectal cancer, nonsteroidal anti-inflammatory drugs, prospective studies

## Abstract

In a prospective cohort study of 74 250 Swedish women and men, with 7.2 years of follow-up and 705 incident colorectal cancer cases, long duration of aspirin use (>20 years) was associated with a reduced risk of colorectal cancer (multivariate rate ratio: 0.65; 95% confidence interval: 0.45–0.94). Aspirin use for a shorter period was not associated with risk.

More than 15 case–control and cohort studies have suggested that aspirin use may lower the risk of colorectal cancer (Bosetti *et al*, 2002). Furthermore, randomised trials have demonstrated that regular use of aspirin reduces the risk of recurrent adenoma within 1–3 years in patients with prior colorectal adenoma or cancer (Baron *et al*, 2003; Benamouzig *et al*, 2003; Sandler *et al*, 2003). However, two other randomised trials that evaluated the effect of aspirin on colorectal cancer did not show a reduction in risk after 5 or 10 years (Gann *et al*, 1993; Cook *et al*, 2005). Therefore, it remains unclear whether aspirin use for a longer time would lower the risk of colorectal cancer. Results from a recent large prospective cohort of US women showed that a significant benefit of aspirin was not evident until more than 10 years of use and with six or more 325-mg aspirin tablets per week (Chan *et al*, 2005).

The purpose of this study was to examine prospectively frequency and duration of aspirin use in relation to colorectal cancer incidence in a population-based cohort study of 74 250 Swedish women and men.

## MATERIALS AND METHODS

### Study population

Two population-based prospective cohort studies of women and men in central Sweden provided data for these analyses: the Swedish Mammography Cohort and the Cohort of Swedish Men. The Swedish Mammography Cohort was established between 1987 and 1990 when all women born between 1914 and 1948 and residing in Västmanland and Uppsala counties received a mailed questionnaire on diet, weight, height, and education. In the autumn of 1997, all 56 030 living participants obtained a new questionnaire that was expanded to include about 350 items concerning diet and other lifestyle factors, medication use (including aspirin), medical history, etc; 39 227 women completed this questionnaire. The Cohort of Swedish Men began in the autumn of 1997 when 48 850 men born between 1918 and 1952 and residing in Västmanland and Örebro counties responded to a mailed questionnaire that was identical to the Swedish Mammography Cohort questionnaire from 1997. Both studies were approved by the ethics committees at the Karolinska Institutet (Stockholm, Sweden) and the Uppsala University Hospital (Uppsala, Sweden).

For these analyses, we excluded those who did not complete the 1997 questionnaire and those who did not provide information on aspirin use. We also excluded those with incorrect or missing national registration number and those with a previous diagnosis of cancer (except nonmelanoma skin cancer). This left 74 250 participants (32 076 women and 42 174 men), aged 45–83 years in 1997, eligible for analyses.

### Assessment of exposures

In 1997, participants completed a questionnaire on demographic characteristics, weight, height, and medical, smoking, dietary, and physical activity history. Participants were asked to report whether they used aspirin or aspirin-containing medicines and, if so, how many aspirin tablets per week and for how many years. In Sweden, the majority of aspirin-containing medicines contain 500 mg aspirin. Data on non-aspirin nonsteroidal anti-inflammatory drugs (NSAIDs) were not available.

### Case ascertainment and follow-up

Incident cases of colorectal adenocarcinomas were identified by computerised linkage to the national and regional Swedish cancer registries, both of which provide nearly 100% case ascertainment in Sweden (Mattsson and Wallgren, 1984). By linkage to the Swedish Death and Population registers at Statistics Sweden, we obtained information on dates of death and migration, when applicable.

### Statistical analysis

Person-time of follow-up was counted for each participant from 1 January 1998 until the date of colorectal cancer diagnosis, death, migration, or 30 June 2005, whichever came first. We used Cox proportional hazards modeling (Cox, 1972) to calculate rate ratios (RRs) with 95% confidence intervals (CIs). Age in months and sex were adjusted for as stratification variables in the model. In multivariate analyses, we further adjusted for education (less than high school, high school graduate, or more than high school), family history of colorectal cancer (yes/no), body mass index (<23.0, 23.0–24.9, 25.0–29.9, or ⩾30 kg m^−2^), leisure-time physical activity (h/week; four categories), history of diabetes (yes/no), and smoking (never, past, or current smoker). Tests of linear trends across categories of aspirin use were conducted by assigning the median value of aspirin use for categories and treating these as a single continuous variable. All analyses were conducted with SAS statistical software, version 9.1 (SAS Institute Inc., Cary, NC, USA). All reported *P*-values are based on two-sided statistical tests.

## RESULTS

At baseline, about 51% of women and 37% of men reported aspirin use. Aspirin users did not differ substantially from nonusers with respect to age, education, family history of colorectal cancer, body mass index, physical activity, diabetes, or smoking status (Table 1).

Among the 74 250 women and men, we ascertained 705 incident cases of colorectal adenocarcinoma (261 women and 444 men) during a mean follow-up of 7.2 years, from 1998 through June 2005. Compared with nonusers, women and men who reported use of more than six aspirin tablets per week had a statistically significant 23% lower risk of colorectal cancer after adjusting for age, sex, and other known and potential risk factors (Table 2). The association was similar for colon and rectal cancers, but appeared to be stronger for distal (multivariate RR, 0.53; 95% CI, 0.30–0.93) than proximal colon cancer (multivariate RR, 0.82; 95% CI, 0.52–1.30). When we examined duration of aspirin use, significant risk reduction was not observed until more than 20 years of use (Table 2). Compared with nonuse, the multivariate RRs of colorectal cancer for more than 20 years of aspirin use were 0.65 (95% CI, 0.45–0.94) in women and men combined, 0.57 (95% CI, 0.30–1.10) in women, and 0.70 (95% CI, 0.45–1.09) in men. Further adjustment for postmenopausal hormone use among women did not change the results materially (multivariate RR, 0.58; 95% CI, 0.30–1.11). The inverse association with long-term aspirin use (>20 years) seemed to be stronger for rectal cancer than for colon cancer (Table 2). In women and men combined, the association between long-term aspirin use and risk of colorectal cancer remained after additional adjustment for intakes of alcohol, red meat, calcium, and folate (all in quintiles) and multivitamin supplement use (multivariate RR, 0.62; 95% CI, 0.42–0.90), and after excluding the first 2 years of follow-up (multivariate RR, 0.65; 95% CI, 0.43–0.98).

## DISCUSSION

In this population-based prospective study of Swedish women and men, long-term (>20 years) aspirin use was associated with a statistically significant 35% reduction in risk of colorectal cancer. Our findings persisted after controlling for other known and potential colorectal cancer risk factors.

Three randomised trials have shown that short-term regular aspirin use reduces the risk of recurrent adenoma in patients with previous polyps or colorectal cancer (Baron *et al*, 2003; Benamouzig *et al*, 2003; Sandler *et al*, 2003). However, findings from the Women's Health Study, a randomised controlled trial including nearly 40 000 women, did not show a reduction in colorectal cancer risk after 10 years of treatment with low-dose aspirin (100 mg on each other day) (Cook *et al*, 2005). Another randomised trial, involving about 22 000 US male physicians, that examined alternate-day use of 325 mg of aspirin found no association with the incidence of colorectal cancer during 5 years of randomised treatment and follow-up (Gann *et al*, 1993). It is possible, however, that the 10- and 5-year intervention periods in these two trials (Gann *et al*, 1993; Cook *et al*, 2005) may have been too short to produce clear effects. Additionally, with regard to the Women's Health Study, the dose may have been too low. In our prospective study, a statistically significant reduction in colorectal cancer incidence was not observed until more than 20 years of aspirin use. Our findings are broadly consistent with those of the Nurses’ Health Study, suggesting that a benefit of aspirin against colorectal cancer may require long-term (>10 years) use (Chan *et al*, 2005). This same study also suggested that the effect of aspirin use on colorectal cancer risk is dose dependent; the lowest dose associated with a statistically significant reduction in risk was 6–14 aspirin tablets (325 mg) per week (Chan *et al*, 2005). Recent findings from the Women's Health Initiative Observational Study (Allison *et al*, 2006) showed no statistically significant association between duration of aspirin use and colorectal cancer risk; the relative risks associated with 10.1–20 years and >20 years of aspirin use were 0.58 (95% CI, 0.24–1.37) and 0.77 (95% CI, 0.40–1.50), respectively, compared with nonuse. The absence of a statistically significant association in the Women's Health Initiative Observational Study may be due to the small number of cases reporting long-term aspirin use (*n*=7 cases for 10.1–20 years and *n*=12 cases for >20 years of use).

The strengths of our study include its prospective and population-based design, the completeness of follow-up of participants through linkage to population-based registers, information on multiple potential confounders, and data on frequency and duration of aspirin use. The prospective design eliminates recall bias and the virtually complete follow-up minimises the possibility that our findings have been affected by differential follow-up of exposed compared to unexposed individuals. Our study is limited by the lack of information on non-aspirin NSAID use. Furthermore, we had limited statistical power to examine the combined effect of frequency and duration of aspirin use on risk of colorectal cancer. Some misclassification of exposure to aspirin is likely to be present in our study. Nondifferential misclassification would most likely result in attenuation of the true relationship between aspirin use and colorectal cancer and therefore is unlikely to explain our findings. Although we adjusted for a wide range of potential confounders, we cannot exclude the possibility that the observed association is attributable to residual or unmeasured confounding.

In summary, our results suggest that long duration of aspirin use may lower the risk of colorectal cancer. The association between long duration of aspirin use and colorectal cancer risk warrants further study.

## Figures and Tables

**Table 1:** Baseline characteristics of the study population by sex and aspirin use^a^

	**Women**		**Men**	
**Characteristic**	**No aspirin use**	**Aspirin use**	**No aspirin use**	**Aspirin use**
Participants (*n*)	15 721	16 355	26 394	15 780
Age, mean (years)	61.2	61.6	59.5	60.8
Postsecondary education (%)	18.0	21.1	16.4	17.5
Family history of colorectal cancer (%)	7.3	8.2	6.8	7.3
Body mass index, mean (kg/m^2^)	25.0	25.1	25.7	26.0
Leisure-time physical activity (h/week)	2.4	2.3	2.6	2.5
History of diabetes (%)	3.5	3.7	5.8	6.5
Current smoker (%)	23.3	23.6	24.5	29.5

a All values (except age) are standardised to the age distribution of the study population at baseline.

**Table 2:** RRs of colorectal cancer by dose and duration of aspirin use in a cohort of 74 250 Swedish women and men, 1998–2005

	**Colorectal cancer**				**Colon cancer**		**Rectal cancer**	
**Aspirin**	**No. of cases^a^**	**Person-years of follow-up**	**Age- and sex-adjusted RR (95% CI)**	**Multivariate RR (95% CI)^b^**	**No. of cases^a^**	**Multivariate RR (95% CI)^b^**	**No. of cases^a^**	**Multivariate RR (95% CI)^b^**
Nonuse^c^	417	304 321	1.00 (reference)	1.00 (reference)	271	1.00 (reference)	149	1.00 (reference)
Use	288	230 610	0.89 (0.76–1.03)	0.87 (0.75–1.01)	185	0.84 (0.69–1.02)	104	0.92 (0.72–1.19)
*No. of tablets/week*								
1	45	42 545	0.85 (0.62–1.16)	0.83 (0.61–1.14)	26	0.73 (0.48–1.09)	20	1.07 (0.67–1.72)
2–6	65	52 524	0.91 (0.70–1.18)	0.88 (0.68–1.16)	38	0.77 (0.55–1.09)	27	1.09 (0.72–1.65)
>6	70	44 954	0.80 (0.62–1.04)	0.77 (0.59–0.99)	41	0.75 (0.55–1.03)	24	0.79 (0.51–1.22)
*P*-value for trend			0.09	0.04		0.05		0.37
*Years of aspirin use*								
1–10	52	81 165	0.98 (0.80–1.19)	0.96 (0.78–1.17)	78	0.88 (0.68–1.13)	50	1.09 (0.79–1.52)
11–20	27	24 901	0.89 (0.60–1.31)	0.87 (0.59–1.29)	19	0.93 (0.58–1.49)	8	0.73 (0.36–1.51)
>20	31	35 481	0.66 (0.46–0.96)	0.65 (0.45–0.94)	23	0.76 (0.49–1.16)	9	0.52 (0.26–1.02)
*P*-value for trend			0.03	0.02		0.20		0.04

CI=confidence interval; RR=rate ratio;

a The number of cases may not sum up to the total number of cases owing to missing information on number of tablets and years of aspirin use. Four cases that were diagnosed with both colon and rectal cancer were included in analysis of both cancer sites.

b Multivariate RRs were adjusted for baseline age, sex, education, family history of colorectal cancer, body mass index, leisure-time physical activity, history of diabetes, and smoking.

c Reference group for all comparisons.
